# Exploring Italian Wine Companies: A Study of Supply Chain Dynamics, Quality, and Promotion Strategies through Semi-Structured Interviews

**DOI:** 10.3390/foods12244429

**Published:** 2023-12-10

**Authors:** Giada Pierli, Fabio Musso, Federica Murmura, Laura Bravi

**Affiliations:** Department of Economics, Society, Politics, Carlo Bo University of Urbino, 61029 Urbino, Italy; g.pierli@campus.uniurb.it (G.P.); fabio.musso@uniurb.it (F.M.); federica.murmura@uniurb.it (F.M.)

**Keywords:** Italian wine companies, food quality, supply chain management, food strategies

## Abstract

In Italy, the wine supply chain is a cornerstone of the national agri-food system and a driving force for the entire economy. The aim of this study is to map the profile of Italian wine companies through the analysis of multiple case studies. The study focuses specifically on companies in the Marche region, investigating their features and supply chain, with a specific focus on product and system certification adopted, marketing policies implemented, and the businesses’ relationship with institutions. A total of 18 companies participated in the study. The primary data for the research was collected through semi-structured interviews lasting 1 h, based on an interview protocol. The results show that the supply chain for the wine sector is configured to be short, even when cooperatives act as an aggregator. The small size and limited financial resources make it particularly difficult to promote the wine outside its reference context, and there is a lack of a network strategy at the local level. Many companies consider organic certification a disadvantage since it tends to homologate production to the large wine industries, making the product’s naturalness, typicality, and craftsmanship disappear.

## 1. Introduction

Wine is among the oldest [1] and most popular alcoholic beverages globally [2], representing a dynamic and relevant market [3,4], with Italy, France, and Spain accounting for 53.0% of the global wine production (49.1, 46.6, and 40.7 mhl, respectively) [3,5].

In Italy, the wine supply chain is one of the cornerstones of the national agri-food system, acting as a driving force for the entire economy and representing, together with pasta, a true symbol of food and wine culture in the world [6,7]. In fact, wine is a country’s heritage and an expression of some fundamental aspects of its history and tradition, and the morphological and climatic characteristics of the area make it possible to create and promote quality viticulture thanks also to the cultivation of over 300 different types of wines, widespread in all Italian regions [8].

Also, considering the consumption issues, Italy represents a market of primary importance in relation to wine, placing itself in third place in the world ranking after the United States and France [5]. In detail, from the sector profile created by Ismea Mercati [9], it emerges that in 2022, Italian wine consumption stood at around 22 million hectoliters, almost equally divided between common wines (11.1 million hectoliters) and wines with geographical indications (11.3 million liters). Like in other countries, a trend toward the estimation of consumption in quantitative terms is also being observed in Italy. However, this decrease is accompanied by an increasingly complex consumption oriented toward the search for characteristic products with a higher quality level [10,11]. In addition to domestic demand, an important contribution to the qualitative growth of Italian wine production also comes from exports, which have strengthened and consolidated this direction. Specifically, in 2019, Italian wine exports amounted to 6.3 billion euros in value, while in terms of quantity, 21.3 million hectoliters of wine were exported; these export flows were mainly destined for countries such as the USA, Germany, and the United Kingdom [5].

As seen from the evolutionary path that has characterized the Italian wine sector, the theme of quality has represented and still represents even more a critical factor for success [12,13].

As far as quality productions are concerned, the Italian territory consists of 408 certified PDO wines and 118 PGI wines, for a total of 526 certified Italian wines (https://www.politicheagricole.it/, accessed on 12 February 2023).

From the study by Di Vita G. et al. [14], some interesting aspects emerged regarding the consumption of wines bearing the European PDO and PGI brands. The first result concerns the predisposition to the consumption of wines with geographical indication. Generally, the consumption of these types of products is, in fact, higher if it takes place outside the domestic context, for example, in places such as restaurants. In addition to this, even the opinion of an expert, such as a sommelier, seems to favor wines with a protected designation of origin and wines with a protected geographical indication [15,16] in consumption choices.

Conversely, when wine is purchased directly from a producer, a geographical indication for the product appears to be of little relevance. In this context, the reputation of the producer is sufficient and has a preponderant weight compared to quality certifications [17]. Finally, a further interesting aspect brought to light through the aforementioned analysis is connected to the distribution channels. In fact, it emerges that modern distribution channels cover a role of primary importance as regards the trade of PDO and PGI wines, given that most of the transactions relating to these products take place, for example, in supermarkets [18].

Furthermore, for wines with a protected geographical indication, an important factor in their sales is attributed to online channels [19]. In more detail, as regards large-scale retail trade, wines with geographical indications cover a share of 55%, in terms of volume, of the total purchases of wine made through this distribution channel. With regard to the value of wine purchases, the share of the total pertaining to wines bearing the geographical indication corresponds to approximately 62% of the total [9].

As far as organic is concerned, in 2020, Italy occupied the first place in the world for the extension of organic vineyards [20]. Specifically, Italy concentrates 24% of the global organic surface, a share slightly below that of France and Spain [21]. Considering national data, Italy has 15% of vineyards managed with the organic method out of the total vineyards [20]. Considering the Marche region, which will be analyzed more directly in this study, the surface area of the region with organic grapes has increased over the years, going from 3287 hectares in 2010 to 5885 hectares in 2019 with a compound annual growth rate of +3.4% in the 2010/14 period and +9.3% in the 2015/19 period. Overall, the percentage of the Marche area devoted to organic grapes represents 5% of the total national vineyard area and 41.9% of the total Marche area, much higher than the national average of 18.8% [22].

Hence, considering the relevance of the Italian wine sector for the national economy and the importance it has abroad, and considering that the Marche region is a relevant territory in Italian wine production, the aim of this study is to map the profile of wine companies, through the analysis of a multiple case study. It focuses specifically on companies in the Marche region, investigating their characteristics and their supply chain, with an upstream focus on suppliers of grapes and winemaking tools, as well as downstream, trying to understand which are the main reference markets for Italian wine and its main distribution channels. Subsequently, specific focuses are placed on quality productions and the role of product and system certifications, the main marketing policies and communication strategies adopted, and the relationship between companies and institutions, while investigating the ideas and future strategies they intend to adopt.

Therefore, the research questions that the paper intends to investigate are the following:

RQ1: How are the Supply Chains of Italian wine companies structured, and how are national and international relationships managed?

RQ2: What is the role of system and product certifications in the development of a quality strategy by Italian wine companies?

RQ3: How are the marketing strategies of Italian wine companies developed and managed, and what is their role in the development of product strategies?

RQ4: What is the relationship of Italian wine companies of the Marche region with the protection consortium and with local institutions, and what strategic interventions do they consider important to encourage their growth?

The value of this paper lies in the great quality and depth of the information obtained through detailed semi-structured interviews, which made it possible to understand the relationships between the actors in the supply chain of Italian wine companies and their internal strategies regarding the promotion and distribution of the wine product in national and international markets, also considering the role played by protection consortia and local institutions in the promotion of these products.

Table 1 presents previous qualitative studies in this area of research to underline the value of our study in covering some aspects not fully covered in previous literature. In fact, previous literature focuses on specific topics such as marketing strategies, supply chain dynamics, and quality or sustainability practices but does not take these elements into consideration as a whole. This is certainly the added value of the qualitative analysis developed.

The rest of the paper is structured as follows: Section 2 defines the methods adopted to develop the multiple case study analysis; Section 3 presents the results of the 18 interviews with Italian wine companies from the Marche region; Section 4 discusses the results obtained; and finally, Section 5 draws the main conclusions.

## 2. Materials and Methods

The research method used for the development of this study is a mixed method based on a procedure in which qualitative and quantitative data were collected in parallel to answer the research questions [37,38], developing an experimental design as suggested by Clark et al. [39].

The methodology of the multiple case study chosen is descriptive, as the research aims to outline the general and particular characteristics of a given phenomenon [40].

The companies for this study were selected according to the following criteria.

Location: all companies must be located in Italy, in the Marche Region;
Industry: all companies must belong to the wine sector.

A total of 18 companies participated in the study.

The primary data for the research was collected through semi-structured interviews, lasting 1 h, based on an interview protocol, with the following contents: the first part presents general questions with the aim of outlining the company and the interviewees’ profiles; the second part analyzes the supply chain of the companies interviewed in terms of supplies, number of suppliers, geographical location, and size of the same, reference markets, and distribution channels; the third part explores the possession of system (ISO 9001, ISO 14001) [41,42] and product certifications (organic certification, PGI, and PDO) of the companies interviewed; the fourth section considers the price and promotion policies adopted by the companies; and the fifth part examines the opinion of the interviewees regarding some strategic interventions to be implemented in the sector. As regards the collection of quantitative data, during the interviews carried out, the interviewees were asked to answer some questions by assigning a numerical score according to a five-point Likert scale (1 = not at all important; 5 = very important) to give a numerical weight to some qualitative statements reported and therefore better evaluate these aspects quantitatively.

Secondary data were gathered thanks to a review of the companies’ websites and profiles on different social networks to comply with the triangulation principle [43] and validate the information obtained through the semi-structured interview.

The paper has two main limitations. First, the study focused only on the Italian Marche region, which is a small Italian region, even if this can be considered among the relevant territories for Italian wine production. Second, the qualitative methodology used does not allow the user to consider a wide sample of companies, even if it has the advantage of obtaining in-depth information on the topic studied.

## 3. Results

### 3.1. Profile of Italian Wine Companies

The first aspect considered during the interviews was that relating to the interviewee’s profile in terms of role, age, and educational qualification. As shown in Table 2, most of the interviewees hold the role of owner (10 out of 18); the remainder carry out important functions, occupying positions of responsibility in the administrative (1), commercial (2), and technical (1) area, as oenologist (1), managing director (2), and president (1). As regards age, the interviewees were, on average, 45.5 years old. Finally, six of them have a high school diploma, five a five-year single-cycle degree, two a first-level master’s degree, one a three-year degree, two a master’s degree, and one a PhD.

Secondly, the study focused on the characteristics of the companies analyzed. In particular, the following elements were investigated: municipality and province of origin; foundation year; legal form; number of members (in the case of a cooperative enterprise); type of management (family or not); number of employed; and turnover range. The data collected during the interviews were confirmed and enriched by a subsequent analysis of the websites and social pages of the companies interviewed.

Table 3 summarizes the collected data. Specifically, the companies analyzed are mainly located in the Italian provinces of Ancona (6), Ascoli Piceno (6), Macerata (5), and Pesaro and Urbino (1). Most of the companies (11) were founded in the mid to late 1990s; the remaining (7) date back to the first decade of the 2000s. In particular, the older company was founded in 1956 (Company 8), while the younger one in 2016 (Company 2).

Overall, the average age of the firms belonging to the sample is 34 years. As regards the cooperatives (Company 12, Company 13, and Company 17), they, respectively, have 360, 150, and 350 members.

The companies investigated are mainly family-run (15 out of 18). Ten of these have fewer than ten employees, and six have between ten and forty-nine employees. The remaining two can be considered medium-sized enterprises, employing fifty-five and sixty-five employees, respectively. Overall, the average size by number of employees of the wineries analyzed amounts to 16.2. The turnover range is medium-low, with ten companies that earn less than one million euros.

Subsequently, the research was focused on the stages of production of raw materials and their subsequent transformation. As far as raw materials are concerned, the investigation focused on grapes, with respect to which the aspects relating to cultivated area (hectares), quantities produced (quintals), and yield (quintals/hectare) were examined.

As shown in Table 4, the average surface area cultivated with grapes by the companies investigated is equal to 137.56 hectares. Firms 12, 17, and 13 are the largest. These are the three cooperative companies participating in the survey. The company with the smallest area cultivated with grapes is Company 10, a micro-enterprise recently founded in the province of Ancona, with three cultivated hectares.

The average quantity of grapes produced is equal to 9772.78 quintals. Firms 17, 13, and 11 rank first in producing grapes; among these are two cooperative companies, which also appeared to have the largest number of cultivated areas, and Company 11, which has a large cultivated area equal to 250 hectares. Firms 2, 3, and 10 produce the smallest quantities of grapes; they are very small family-run businesses characterized by small cultivated areas, with 560, 500, and 200 quintals of grapes produced, respectively.

As far as the yield is concerned, the average is 92.39 quintals/hectare, but there are considerable differences, going from 150 quintals/hectare of Company 17 to 50 quintals/hectare of Company 5.

The quantities of grapes transformed into wine are, on average, equal to 53,869 hectoliters and 563,778 bottles (Table 4). This underlines a type of production that focuses not on quantity but on the quality of the wine produced, developing product niche strategies with fine wines and important “reserve” bottles. As regards the bottles produced, companies 8 (3,000,000 bottles), 11 (2,000,000 bottles), and 13 (1,200,000 bottles) have the highest production.

The main types of grape variety grown are Montepulciano (in 13 companies), Sangiovese (in 10 companies), Verdicchio (in 10 companies), Passerina (in 7 companies), and Pecorino (in 7 companies); this result indicates that the companies interviewed in the Marche region have a general prevalence of white grape varieties compared to the black ones. The less cultivated ones include Lacrima, Ribona, Grechetto, Sauvignon, Merlot, Pinot, Moscato, Trebbiano, Bianchello del Metauro, Chardonnay, and Grenache.

As regards the seasonality of sales, some of the companies that sell most to the Ho.Re.Ca channel and to intermediaries/wholesalers indicated that the periods of greatest sale of their products are those prior to the summer holidays and Christmas holidays, while those who make use of direct sales to the final consumer indicate the periods of greatest sales in the summer months and in December.

### 3.2. The Upstream and Downstream Supply Chain and the Main Relationships among Intermediaries

In order to respond to RQ1, in the third part of the interview, other aspects of the Italian wine supply chain were explored, both for the upstream and downstream phases.

The suppliers of winemaking products and bottles are mainly located in Italy, while the corks are purchased locally, nationally (especially from Sardinia), and internationally (especially from Portugal). The local supply of labels and cartons mainly comes from companies in the province or the municipalities bordering the same companies interviewed.

Firms supplying winemaking products, corks, and bottles are mainly large, while those supplying cartons and labels are mainly medium and small. Based on the indications of the interviewees, it is also possible to note that many companies interviewed use a single supplier for each of the products indicated.

The number of companies that use vertical or horizontal integration tools (4), signaled by the presence of specific contractual forms, is limited. In particular, Companies 1 and 8 have cultivation contracts, Company 12 has cooperation contracts, and Company 18 has grape supply contracts.

As regards the phases of downstream supply chain (Table 5), the companies interviewed operate on all types of markets, from the regional one to the extra-European one. All companies are present in the national market. Only Company 2 does not operate in the European market; 16 of them are present in the non-European market, and 14 are in the regional market. It is therefore important to point out that four companies (in particular, Companies 2, 3, 5, and 7) exclusively sell their wine to markets outside the region. On average, the largest share of turnover of each company derives from the national market (38.33% on average), followed by the regional one (32.75%), the European one (14.53%), and the extra-European one (14.39%).

As far as the European market is concerned, the main recipient countries of Marche wine are Holland, Belgium, and Great Britain, while the United States, Canada, and Japan are the largest non-European importers.

The distribution channels most used on the national market are Ho.Re.Ca, direct sales, and large-scale distribution. On average, 50.69% of the turnover of the companies analyzed is generated through the Ho.Re.Ca channel, 16.97% through direct sales, and 15.03% through large-scale distribution (Table 6). Direct sales greatly affect the turnover of Companies, which more often use different types of packaging than bottles, such as bags in boxes.

Finally, companies in the sample that present the highest number of bottles produced (from 300,000 to 2,000,000) derive their turnover mostly from large-scale distribution, confirming the fact that to sell to large-scale distribution, it is necessary to have a substantial production capacity, which must however also be associated with a substantial organizational capacity for being able to support the relationship efficiently and in compliance with the requirements set by retailers. Finally, it is noted that all the companies investigated reach foreign markets through the exclusive use of intermediaries/wholesalers, with the exception of Company 1, which obtains about 10% of its foreign turnover also through online sales, and of Company 12, which obtains 60% of its foreign turnover by turning directly to the foreign Ho.Re.Ca channel.

### 3.3. System and Product Certification

The fourth section of the interview aims to provide an overview of the certifications held by the companies examined, obtaining information to answer RQ2. All companies possess the mandatory system certification, Hazard Analysis and Critical Control Points (HACCP), while for the voluntary ones, the situation is different (Table 7). In fact, only Companies 8, 13, and 17 hold the British Retail Consortium (BRC), International Food Standard (IFS), Equalitas, and ISO 9001 certifications. On the other hand, the organic certification is present in almost all companies (16 out of 18) for at least 11 years (on average). Company 5 (31 years), Company 9 (22 years), Company 12 (22 years), and Company 8 (17 years) are the ones that have been involved in this type of certification for the longest time.

On average, the percentage of turnover derived from organic products is 54.63%; for Companies 4, 5, 7, 10, and 14, this percentage is 100%.

The area cultivated with organic farming is 100% in most of the companies interviewed. The statements of Companies 2 and 3 are interesting, as they show contrasting opinions with respect to the concept of organic certification despite the similarities of their company profile (Table 7). The first, in fact, considers the certification process challenging but, at the same time, essential to validate and demonstrate its link with the culture and tradition of the Marche region in Italy. On the other hand, the second fully recognizes the value of organic production and affirms that its corporate philosophy is completely oriented in this direction; however, the certification path is bureaucratically inaccessible. In response to this difficulty, the company has decided to demonstrate the naturalness and genuineness of the wine produced without making use of the organic certification but strongly communicates the authenticity of its work. To this end, the owner has started collaborations with laboratories and universities to prove the quality requirements of his wine, which shows particular features in the fermentation phase.

All the interviewees hold additional product certifications, such as PGI and PDO, unanimously emphasizing their fundamental importance for competitiveness within the sector. The latter aspect is further highlighted by the continuity of these certifications in the companies observed, which have been certified on average for 34.5 years. In addition, it is observed from the budget documents that the companies have shared with us that the percentage of turnover generated by PGI and PDO-certified products is equal to 100% in most cases analyzed.

Interesting considerations emerged from the discussion with the producers interviewed on these aspects. As regards the European certifications relating to the denomination of origin (PGI and PDO), the decision to certify the production is made necessary by the need to be able to compete successfully on the market, as the only products that do not inevitably have to possess the certification are those not bottled, sold in bulk. Regarding organic certification, on the other hand, many companies consider this certification to be a disadvantage despite having it since it tends to homologate production to the large wine industries, making the naturalness, typicality, and craftsmanship of the Italian product disappear.

### 3.4. Marketing Policies

A part of the interview was dedicated to the price and promotion policies adopted by the companies in the sample (Table 8) to understand their role in the development of product strategies (RQ3). The whole sample stated that they have product lines with basic prices and premium wines, often “reserva” wines, which are sold at higher prices (up to 70 euros per bottle). Regarding the weight of some elements in the definition of the selling prices of the products, it emerged that for entry-level wines, the company costs are very important (4.19 euros on average on a scale from 1 to 5), which are preponderantly compared to the brand (3.06). However, for premium wines, the choices focus almost exclusively on the positioning and reputation of the brand to define the price of the bottle. Furthermore, it emerged that the brand has a much higher value than any certification held, such as the organic one, in defining the price.

It should be noted that, in any case, for basic and premium products, entrepreneurs attach little importance to the price of competitors since, especially for premium lines, many believe they are unique in their production, so much so that they consider not having direct competitors.

As regards the increase in the price of wine following the acquisition of organic and/or product certification, the interviewees show uneven opinions. In fact, 10 of them agree that obtaining the certification has allowed the price to rise in response to an increase in the value of the product. On the other hand, the other interviewees declare that they have not increased the price of the certified product, including Company 6, which considers the certification completely irrelevant to the price of its wine due to the reduced perception of Verdicchio’s value on the market.

All companies recognize the importance of carrying out marketing activities, even if some are still little evolved. For example, Company 4 stated that it focused mainly on improving the production process, but the willingness to invest in marketing activities is certainly planned.

In general, the management of marketing operations is predominantly carried out within the company, with only six companies that have completely outsourced this activity.

The importance of carrying out marketing actions for the enhancement of typical and certified products is recognized overall but does not play, for the entrepreneurs interviewed, a central role in the promotion of the product. What is considered important is the ability to convey the passion and values embodied in the making of wine, considering certification (especially product certification) a necessity to compete with large industrial groups rather than a real proof of quality. In this regard, Company 10 reiterates that its strategy is mainly based on the naturalness and authenticity of its wine, as product certification is not always positively perceived by consumers.

In general, therefore, the possession of the certification is considered an aspect with a limited influence on the notoriety of one’s brand, just as the search for reaching new consumer segments is of little relevance. Only Company 4 makes a clarification in this regard, specifying that the organic certification assumes an important weight. Also, considering the relationship with new geographical markets, most companies believe the certification has not affected this element.

Finally, as far as promotional activities are concerned, almost all companies usually organize events for tourism purposes, bringing the public closer to their product through tastings, visits to cellars, and cultural events.

Subsequently, the entrepreneurs were asked which were the most used communication channels for the promotion and enhancement of their products (Table 9). The results of the interviews showed that all companies have a website and use social media. Similarly, all the entrepreneurs interviewed, with the exception of Company 14, declare that they participate in fairs and events. In particular, six companies participate in events only in Italy, and ten companies also participate abroad.

Among the communication tools used, specialized magazines and industry guides stand out above all, while only in very few cases are media such as radio, TV, and the press used. The annual budget spent on communication and exhibitions (especially in Italy) is substantially proportional to the turnover, with percentages ranging from 2–3% to 5–10% of the annual turnover.

### 3.5. Institutions, Associations, and Development Paths

The final section of the interview focused on the relationship with the protection consortium and local institutions and on the importance of possible strategic interventions to encourage the growth of the Marche wineries (RQ4). In general, it emerges that the relationship with the protection consortium is considered quite important by all the companies interviewed. However, most of them report a lack of support for the needs and interests of small producers due to a prevailing tendency to facilitate larger companies. Some interviewees feel the need to implement structural reform of the Consortium to obtain a more equal and homogeneous representation among the various members. Similarly, the relationship with local public institutions is considered very important by the interviewees, as they consider it a fundamental tool for seizing the opportunities offered by agricultural policies, such as access to specific tenders and funding. For example, most of the companies have obtained the funds provided by the Regional Development Plan (PSR), making important improvements to their business. Furthermore, some interviewees underline the closeness and sensitivity of the Marche region towards wine producers, recognizing their value and strategic importance for the territory.

Although the relationship with public institutions is assessed favorably overall, some suggestions emerge for the future. Various companies consider the preparation of ad hoc promotion policies to enhance the value of Marche wine as crucial. In this sense, the interviewees highlight the need to make significant investments in food and wine tourism, with the aim of spreading and increasing the value of the territory and its specificities.

Regarding the importance of implementing specific strategic interventions for the future, the interviewees showed themselves to be quite compact in judging the actions in favor of environmental sustainability, those aimed at integration with tourist activities, as well as collaborations with universities and research. With respect to the issue of environmental sustainability, some companies have stated that they are constantly committed to this direction (for example, Company 9 has been preparing the sustainability report for 5 years and intends to obtain B-corp certification), while other companies need greater support from local public institutions. In fact, Company 6 manifests the need for more substantial interventions for the management of climate change, especially in terms of support for irrigation activities, which are now completely the responsibility of companies. Similarly, Company 5 argues for the need for greater incentives to favor the ecological transition, highlighting the economic difficulties of firms in implementing this transformation with total autonomy.

In relation to the integration with the tourist activities of the area, all the companies reiterate the need for structured interventions by the region. Companies 3, 5, and 13 point out the importance of facilitating the creation of a link with the various operators in the tourism sector to compensate for the substantial lack of cooperation. The absence of collaboration has prompted some companies (10 and 18) to seek partnerships with tour operators independently, but they have encountered enormous difficulties. The opinion of Company 6 is also significant, which feels the need to stimulate the tourist flow in order not to make interventions to strengthen the attractiveness of wineries superfluous. Company 16 is also in line with this position, underlining the absence of luxury accommodation facilities and, consequently, the reduced possibility of promoting one’s wine, aimed at demanding consumers with high spending power.

In terms of collaboration with universities and research centers, all the companies interviewed declare themselves very inclined to maintain or activate collaboration paths in the awareness of the positive effects in terms of growth opportunities. Some realities, such as Companies 2 and 13, declare that they already have forms of cooperation underway with the universities of the Marche region, while others promote internship activities in collaboration with higher institutes but are inclined to extend the collaboration with academic realities and research.

In relation to the opening of new distribution channels and the development of foreign markets, Company 3 highlights the great importance of developing an institutional marketing system capable of preparing initiatives for stimulating and supporting wine producers, currently lacking in setting up and carrying out these activities.

## 4. Discussion

The objective of this study was to analyze Italian wine companies, focusing on the Marche region, which represents an essential dimension of the Italian wine production sector, significantly contributing to the enhancement of the territory both nationally and internationally. The research focused on 18 wine companies, analyzing not only their organizational and structural peculiarities but also their strategies for promoting Italian wines. The entrepreneurs’ opinions on the main needs and requirements of the local realities are also analyzed. Answering RQ1, it can be said that these businesses are predominantly family-run and are largely micro and small in size, factors that further support the sense of uniqueness and strong belonging to the territory, an aspect that constantly emerged during the conduction of the interviews. These are contrasted with a few large cooperatives that bring together minor producers who do not have autonomous winemaking structures.

The supply chain characterizing the wine sector is, in any case, configured as a short supply chain, even in cases in which the cooperative acts as an aggregator. Upstream of the companies observed are suppliers of capital goods, products for cultivation and winemaking, and packaging materials (caps, bottles, labels, and packaging), with a number that tends to be limited to one or a few suppliers for each type of purchase.

The orientation of the entrepreneurs is to make use of local or national suppliers in cases where local procurement is more difficult. Only in rare cases is there recourse to foreign suppliers, generally if there is a lack of suitable suppliers in the national territory.

Downstream of the manufacturing companies, the market in Italy is made up of the Ho.Re.Ca channel, which, in the case of the panel of companies interviewed, absorbs 50.69% of the turnover. Direct sales account for 16.97% of turnover, and 15.03% goes through large-scale distribution, with the remainder being divided between wholesalers and online sales. What most differentiates the smallest businesses from the more structured ones (above all cooperatives) is the ability to relate to modern channels, i.e., the large-scale distribution, to which small producers have no access capacity, nor they often look for it having chosen a positioning that differs from the price comparison, to which the relationship with the DGO exposes.

In the relationship with foreign markets, in almost all cases, recourse is made to importers, with whom collaborations are often the result of contacts made during national or international trade fairs. However, relationships developed directly with wine bars or restaurateurs are not uncommon following reports from tourists who, upon returning home, take steps to find suppliers from whom to source the wines appreciated during their stay in Italy. This phenomenon is giving rise to a mechanism of relationships that, in a widespread way, feeds micro-networks of commercial relationships capable of sustaining and replicating themselves, generating the conditions to encourage entrepreneurs towards more decisive and active commercial actions in the identified markets [44].

A limitation that emerges within the context of the supply chains to which they belong is that these are, in fact, not very capable of generating virtuous mechanisms linked to aggregation dynamics. If we exclude the cooperatives, whose function is precisely that of creating a critical mass on various fronts, the companies that carry out the entire cultivation/vinification cycle organize the relationships both upstream and downstream individually without looking too much for collaborations horizontally. Only recently have there been attempts to aggregate promotional efforts to increase the visibility of certain types of wine, as also suggested by Dolan and Goodman [45].

Product certifications are considered no longer as an element of added value but as a necessary and basic element to be able to sell their wines. In this sense, some of the entrepreneurs interviewed are keen to underline that their name is even stronger than the possession of organic certification, which, on the contrary, risks leading to homologation with large industrial groups rather than being a tool for further support of the naturalness, typicality, territoriality of its product. This is in line with what has been found in the literature: sometimes, the producer’s reputation is sufficient and has a preponderant weight compared to the quality certifications [17]. However, almost all the companies have organic certification, even if the cost and the greater weight of the procedures to be followed are sometimes complained of, as it represents an identity card demonstrating the quality of the product made-especially in international markets where the name of the individual producer is almost unknown (RQ2).

Answering RQ3, the small size and limited financial resources make promoting the wine outside its reference context particularly difficult. The producers interviewed highlight the lack of a network at the local level that facilitates the diffusion of the product on the market and enhances the varieties of Marche wine, developing strategies like those successfully implemented for wines from other regions (especially with reference to Veneto). This limit is also due to the fact that producers show a tendency to concentrate mainly on improving the production process, to the detriment of promotion and communication strategies. In fact, in the case of smaller companies, marketing is managed directly by the owner, as well as many other functions, such as sales and oenologist. In larger companies, there is a greater structuring in the performance of marketing activities, sometimes also through external agencies for communication, albeit always in a partial and limited way.

The promotional activities in which companies invest the most are represented by participation in fairs and events, but the organization of initiatives at the company headquarters is also growing, including theme nights, demonstrations, food and wine events, and guided tours.

On the promotional front, the producers openly declare the need for support from the protection consortium and the local public institutions. With regard to the first, the need for support in carrying out marketing activities is underlined, especially in terms of national and international promotion, and some entrepreneurs report that they already have ongoing collaborations with regard to support for trade fairs, especially in extra EU countries. As far as the public institutions of the area are concerned, the preparation of ad hoc promotion policies for the valorization of Marche wine is considered crucial. In this sense, the interviewees highlight the need to implement important investments in food and wine tourism, with the aim of spreading and increasing the value of the territory and its specificities.

Answering RQ4, from the interviews, the relevance of investing in tourism with a multi-year project that goes beyond the deadlines of the administrations emerges with the aim of intercepting both foreign and Italian tourist flows. At the same time, however, producers believe that the individuality of each company, its products, and its name must be maintained. From this point of view, they do not consider it necessary to integrate themselves into consortia or cooperatives since this would overshadow their identity, which is considered their main strength, more than having a product certification such as PDO or PGI.

Other elements that emerge from the study concern the ability to adapt to changes in the competitive scenario. This ability has repercussions on innovation processes, which for the sector means knowing how to combine tradition and innovation. The innovation concerned not only the cultivation and winemaking techniques but also the business models themselves, leading over the years to the opening up to the incoming tourist market and the development of tourist attraction initiatives, and through organizing hospitality structures (agritourism, resorts, and B&Bs). Another front of change has been in expanding the range of products offered via exploiting the complementarities of supply in local food production. This generates forms of connection and interdependence with local operators in other sectors, such as producers of preserves based on local products, cheeses, oil, and pasta. Similarly, collaboration with tour operators (travel agencies and hotels) is growing, with the aim of enhancing the attractiveness of the area and integrating the tourist offer with that linked to history, art, culture, and local traditions.

The dynamics of the tourism sector have influenced the business model of wine producers and, at the same time, the presence of specialized wine productions has made it possible to modify the local tourist offer, according to a mutualistic co-evolution scheme [46], which it can be observed when organizations develop capacity for cooperation and complement each other. Over the years, the characterization of the local tourist destination has, in fact, changed from the summer-seaside focus to becoming increasingly attractive to tourists throughout the year, with a much wider value offer than the seaside themes.

Internationalization was another relevant change in strategy. The opening to foreign markets took place mostly from the 2000s, often relying on the occasional personal contacts or connections provided by tourists, then turning to restaurants, small importers, or individual wine bars, not relying on networks as relevant tools defined in the literature [36].

In light of the results that emerged from the study, it is possible to draw a summary picture from which to draw indications regarding the strategies to be adopted to strengthen the Italian wine production system and integrate it more within the prospects of relaunching and growing the national economy. From the resulting SWOT analysis (Table 10), the presence of strong potential appears evident, both for the characteristics of the productions made and for the considered context, particularly suitable for favoring the connotation of the wine offer in an integrated way with other enhancement elements of the economy.

The link with tourist flows represents the strong element that characterizes the wine supply chain in the territory analyzed because it is still a front full of potential, which is, moreover, able to favor significant processes of mutual contamination between tourism and food and wine. In the region, tourism is developing, both in the summer season and for the historical, artistic, and architectural heritage of the hinterland, which is rich in medieval and Renaissance villages and castles. The relationship with this heritage is particularly close when wine and typical products are identified with quality labels and marks, which protect their identity and are attributed to those products whose characteristics depend on the territory in which they are produced. What makes this characterization still weak, however, is the identity of the region, which still has to position itself decisively with respect to a national framework that sees the presence of areas with highly incisive identities (for example, Tuscany and Salento). The fact that there are more regional wines potentially able to successfully establish themselves on the market, not just the national one, paradoxically becomes a limit because it prevents the achievement of a strongly defined recognition, as in the case of prosecco for the Veneto or Chianti for Tuscany.

This problem intersects with the identity of the individual producers who, apart from the leading cooperatives at the regional level that affirm their brand, claim their autonomy by pursuing distinctly individualistic niche strategies, but at the same time, feel the need to mass critical for the promotion and development of markets. Consequently, the connecting function that can reconcile substantially divergent needs can only be that of public and institutional subjects capable of identifying a shared strategic path but capable of affirming the identity that the regional wine sector still lacks.

## 5. Conclusions

The lines to be followed to favor an evolution path capable of maximizing the potential of the sector and reconciling the expectations of individual companies are summarized below. In this framework, the broader context should not be overlooked—if we talk about harmony between qualified wine supply and quality tourism with which to create synergies—when the offer must be adequate to the characteristics of the demand to which it is addressed. This means that the accommodation facilities must be adequately qualified, the infrastructures efficient, and the public and private services up to a demand that, if qualified and with a high spending capacity, cannot give up minimum levels of quality in general hospitality.

The lines of intervention to be considered are the following:

- Promote the wine offer as a regional offer (Marche wines) rather than the various wines individually (Piedmont model) through fairs and other forms of promotion both in Italy and abroad;
- Stimulate cross-sector projects for the promotion and enhancement of wine producers, producers of agri-food specialties, and tour operators (accommodation facilities and incoming operators);
- Stimulate quality policies in wine production, going beyond the mere biological connotation;
- Move the fulcrum of interventions from alliances (network contracts, etc.) to projects;
- Support continuous training activities aimed at entrepreneurs to improve managerial and marketing skills;
- Introduce targeted interventions for the qualification of widespread accommodation facilities;
- Introduce targeted interventions in the context (wine roads, signs, etc.);
- Stimulate entrepreneurship in the sector and agro-tourism.

The main limitation of the study derives from the fact that it is focused only on one Italian region, which is Marche, even if the Italian regional realities are very similar and could be easily compared to Marche among the relevant national territories for Italian wine production. In any case, for future research, it could be interesting to develop the same study nationally in order to verify similarities and differences among different regions. It would also be interesting to compare the Italian wine sector with other European realities to make a comparison of similar and different elements of Italian and European wine companies and their strategic behaviors in the sector. Another limitation derives from the fact that qualitative methodologies do not allow consideration of a wide sample of companies, even if they have the advantage of obtaining in-depth information on the topic. In order to overcome this limit, it would be interesting in future research to combine these results with a survey carried out on a larger sample of Italian companies in the sector.

## Figures and Tables

**Table 1:** Previous qualitative studies on wine companies and their economic behavior in the last ten years.

Authors	Year	Title
Szolnoki, G.	2013	A cross-national comparison of sustainability in the wine industry [23]
Giacosa, E., Giovando, G., Alberto, M.	2014	Wine sector as a driver of growth for the Italian economy [24]
Garcia-Galan, M. M., del Moral-Agundez, A., Galera-Casquet, C.	2014	Valuation and importance of the extrinsic attributes of the product from the firm’s perspective in a Spanish wine-protected designation of origin [25]
Covarrubias, J., Thach, L.	2015	Wines of Baja Mexico: A qualitative study examining viticulture, enology, and marketing practices [26]
Vrontis, D., Bresciani, S., Giacosa, E.	2016	Tradition and innovation in Italian wine family businesses [8]
Murínová, A.	2017	Wine Marketing: The Case of Micro and Small Wine Companies in the Czech Republic [27]
Dominici, A., Boncinelli, F., Marone, E.	2019	Lifestyle entrepreneurs in winemaking: An exploratory qualitative analysis on the non-pecuniary benefits [28]
Felzensztein, C., Deans, K. R., Dana, L. P.	2019	Small firms in regional clusters: Local networks and internationalization in the Southern Hemisphere [29]
Vergamini, D., Bartolini, F., Prosperi, P., Brunori, G.	2019	Explaining regional dynamics of marketing strategies: The experience of the Tuscan wine producers [30]
Canovi, M., Pucciarelli, F.	2019	Social media marketing in wine tourism: Winery owners’ perceptions [31]
Calderón, H., Fayos, T., Frasquet, M.	2020	The transition of small Spanish wineries toward multi-channel distribution: the role of ambidexterity [32]
Cuel, R., Cangelosi, G. M.	2020	In vino veritas? Blockchain preliminary effects on Italian wine SMEs [33]
Rabadan, A.	2021	Consumer Attitudes towards Technological Innovation in a Traditional Food Product: The Case of Wine [34]
Obermayer, N., Kővári, E., Leinonen, J., Bak, G., Valeri, M.	2022	How social media practices shape family business performance: the wine industry case study [35]
Franco, M., Martins, R.	2023	The role of networks in the internationalization process of small and medium-sized enterprises in the wine-producing sector [36]

**Table 2:** Profile of interviewees.

Company	Role	Age (Years)	Degree
1	Owner	=	=
2	Owner	39	Master
3	Owner	46	Five-year single-cycle degree
4	Owner	37	High school graduation
5	Owner	68	High school graduation
6	Oenologist	43	Five-year single-cycle degree
7	Owner	36	Bachelor degree
8	Chief Executive Officer and President	56	Five-year single-cycle degree
9	CEO	54	Five-year single-cycle degree
10	Owner	46	Five-year single-cycle degree
11	Administrative manager	33	Five-year single-cycle degree
12	Export manager	30	PhD
13	President	66	High school graduation
14	Owner	32	High school graduation
15	Owner	53	Five-year single-cycle degree
16	Owner	41	High school graduation
17	Commercial employee	35	Master
18	Technical director	60	High school graduation

**Table 3:** Company profiles.

Company	Municipality Province	Foundation Year	Legal Form	Partners (If Coop.)	Family Run	Number of Employee	Turnover-€ (2021)
1	Fratte Rosa (PU)	2007	S.s.	NO	YES	10	700,000
2	Apiro (MC)	2016	S.r.l.	NO	YES	3	≤500,000
3	Carassai (AP)	2006	S.r.l.	NO	YES	2	≤500,000
4	Colmurano (MC)	1962	S.s.	NO	YES	11	500,000–1 Mln
5	Offida (AP)	1979	S.s.	NO	NO	8	≤500,000
6	Jesi (AN)	1998	S.r.l.	NO	NO	10 + 5 seas.	500.000–1 Mln
7	Montefiore dell’Aso (AP)	2005	S.s.	NO	YES	9	1 Mln–3 Mln
8	Osimo (AN)	1956	S.p.A.	NO	YES	65	11 Mln–50 Mln
9	Montefano (MC)	2000	S.s.	NO	NO	40 + 15 seas.	3 Mln–5 Mln
10	Senigallia (AN)	2010	S.s.	NO	YES	4	≤500,000
11	Monsampolo del T. (AP)	2008	S.r.l.	NO	NO	8	1 Mln–3 Mln
12	Ripatransone (AP)	1969	Coop.	360	NO	26	5 Mln–10 Mln
13	Matelica (MC)	1971	Coop.	150	NO	18 + 10 seas.	3 Mln–5 Mln
14	Staffolo (AN)	1992	S.s.	NO	YES	4	500,000–1 Mln
15	Jesi (AN)	1968	S.r.l.	NO	YES	15	500,000–1 Mln
16	Apiro (MC)	1978	S.s.	NO	YES	3	500,000–1 Mln
17	Castignano (AP)	1960	Coop.	350	NO	20	3 Mln–5 Mln
18	Serra dei Conti (AN)	1995	S.r.l.	NO	NO	8	3 Mln–5 Mln

**Table 4:** Raw materials and processed products.

	Raw Materials (Grapes)			Processed Product (Wine)			
Company	Cultivated Area (ha)	Produced Quantities (q)	Yield (q/ha)	Processed Quantities (hl)	Bottles (n.)	Cultivated Vines	Months of Highest Sales
1	15	1350	66	1000	135,000	Bianchello del Metauro DOC, Pergola DOC Aleatico, Colli Pesaresi DOC, Sangiovese	April–June
2	8	560	70	500	33,000	Verdicchio	March May–July November
3	5	500	100	150	20,000	Pecorino, Passerina, Sangiovese, Montepulciano	May–September
4	23	2100	100	1500	90,000	Maceratino Ribona, Sangiovese, Montepulciano, Lacrima, Passerina, Chardonnay, Sauvignon, Merlot	June–July December
5	11	600	60	450	50,000	Montepulciano, Pecorino, Trebbiano, Sangiovese, Passerina, Morettone	April–May November–December
6	33	3000	90	2300	150,000	Verdicchio, Montepulciano	March–September
7	50	6000 produced 2000 purchased	120	6000	250,000 bottles 3600 bag in box	Sangiovese, Montepuliciano, Trebbiano, Passerina, Pecorino	July, August, December
8	200	18,000	90		3,000,000	Verdicchio, Pecorino, Montepulciano, San Giovese, Sauvignon, Merlot	March, April, July, October, November
9	120	12,000	100	10,500	300,000 bottles 200,000 bag in box	Ribona, Montepulciano, San Giovese, Grechetto, Verdicchio, Chardonnay, Sauvignon Blanc, Pinot Grigio, Merlot, Cabernet Sauvignon	October–December
10	3	200	70	140	25,000	Verdicchio, Sangiovese, Lacrima di Morro d’Alba	May–September (B2B) August, December (B2C)
11	250	25,000	100	15,000	2,000,000	Passerina, Pecorino, Sangiovese, Trebbiano, Montepulciano, Merlot, Cabernet, Chardonnay	July–September
12	900	7500	83	N,I	1,000,000	Passerina, Pecorino, Sangiovese, Trebbiano, Montepulciano	July–September
13	50 owned 300 of partners	28,000	80	22,000	1,200,000	Verdicchio, Colli maceratesi	May–September
14	14	1000	70	65,000	700,000 bottels 200,000 in bag in box	Verdicchio, Trebbiano, Montepulciano	April–August
15	47	5000	106	3500	150,000	Verdicchio, Sauvignon, Moscato, Montepulciano, Grenache	May–July November–December
16	13	1300	100		80,000	Verdicchio	March–June
17	400	60,000	150	40,000	600,000	Trebbiano, Passerina, Pecorino, Montepulciano, Sangiovese, Merlot, Moscato, Cabernet-Sauvignon	August December
18	35	3800	108	5000	1,000,000	Verdicchio, Montepulciano, Sangiovese	March–August
Total	2476.00	175,910.00		808,040.00	10,148,000.00	-	-
Mean	137.56	9772.78	92.39	53,869.33	563,777.78	-	-

Company 8 specified that this number refers to the total number of bottles that are obtained from their own wine production and the hectoliters purchased.

**Table 5:** The downstream supply chain of interviewed companies.

Company	Markets (% Income)			
	Regional	National	European	Extra-European
1	(40%)	(27.5%)	(25%) France, Holland, Germany, Poland	(7.5%) Japan, United States
2	-	(75%)	-	(25%) United States
3	-	(80%)	(5%)	(15%) United States
4	(50%)	(30%) Centro-Nord Italia	(20%) Belgium, Holland, Albania, Poland	-
5	-	(70%)	(10%) Great Britain, Denmark, Belgium, France	(20%) United States, Canada
6	(42%)	(28%)	(24%) Germany, Belgium, Holland and Great Britain	(6%) United States
7	-	(70%)	(15%) Belgium, Holland	(15%) China, Russia, America
8	(10.5%)	(24.5%)	(32.5%)	(32.5%) Canada, Japan, United States, South Korea
9	(77%)	(10%)	(5%)	(3.25%) Mexico, United States, Canada (4.75%) Asia
10	(30%)	(30%)	(10%) Great Britain, Switzerland, Holland, Czech Republic	(30%)China
11	60%	20%	10%	10% (5%) America, United States, Canada, Mexico(5%) Asia
12	(40%)	(52%)	(3%)	(2%) United States, Mexico, Canada, (3%) Asia
13	(30%)	(50%)	(15%) Great Britain, Germany, Belgium, Holland, Greece	(5%) North America, China, Japan, Russia
14	(25%)	(25%)	(25%) France, Spain, Sweden, Denmark, Holland, Belgium	(25%) United States, Canada, South Korea
15	(60%)	(25%)	(10%)	(5%) United States
16	(20%)	(20%)	(30%)	(30%) United States
17	(75%)	(13%)	(12%) Norway	-
18	(30%)	(40%)	(10%)	(20%) United States, Japan
Mean	28.19%	35.25%	13.19%	14.06%

**Table 6:** The distribution channels of interviewed companies.

	National Market (% Turnover for Each Channel)					Foreign Market (% Turnover for Each Channel)				
Company	Online	Direct Sale	Ho.Re.Ca.	Intermediaries/Wholesalers	Large Distribution	Online	Direct Sale	Ho.Re.Ca.	Intermediaries/Wholesalers	Large Distribution
1	10	15–20	50–55	-	10–15	10	-	-	90	-
2	-	5	15	-	-	-	-	-	100	-
3	-	25	25	-	-	-	-	-	100	-
4	-	20	80	-	-	-	-	-	100	-
5	-	10	60	-	-	-	-	-	100	-
6	-	20	80	-	-	-	-	-	100	-
7	-	40	10	-	20	-	-	-	100	-
8	-	-	75	-	15	-	-	-	100	-
9	-	20	5	-	45	-	-	-	100	-
10	-	10	90	-	-	-	-	-	100	-
11	2	5	20	13	60	-	-	-	100	-
12	2	25	45	20	8	-	-	60	40	-
13	-	20	50	-	30	-	-	-	100	-
14	10	-	90	-	-	-	-	-	100	-
15	2	33	65	-	-	-	-	-	100	-
16	-	-	100	-	-	-	-	-	100	-
17	1	45	25	9	20	-	-	-	100	-
18	5	10	25	-	60	-	-	-	100	-

**Table 7:** Product and system certification tools.

Company	System Certification	Type	Organic Certification	Years of Possession	Organic Cultivated Area (%)	Organic Products Turnover (%)	Other Product Certifications	Type	Years of Possession	Product Certification Turnover (%)
1	YES	HACCP	YES	2	100	90	YES	PGI PDO	15	90
2	YES	HACCP	YES	6	100	85	YES	PDO	6	95
3	YES	HACCP	NO	N.I.	N.I.	N.I.	YES	PDO	11	100
4	YES	HACCP	YES	5	100	100	YES	PDO	53	100
5	YES	HACCP	YES	31	100	100	YES	PGI PDO	43 for PDO 30 for PGI	100
6	YES	HACCP	YES	5	100	10	YES	PGI PDO	24	100
7	YES	HACCP	YES	13	100	100	YES	PGI PDO	17	60
8	YES	HACCP, BRC, IFS, Equalitas	YES	17	100	30	YES	PGI PDO	43 for PDO 30 for PGI	98
9	YES	HACCP	YES	22	50	20	YES	PGI PDO	22	100
10	YES	HACCP	YES	8	100	100	YES	PGI PDO	12	100
11	YES	HACCP	YES	10	60	60	YES	PGI PDO	N.I	N.I
12	YES	HACCP	YES	22	50	14,50	YES	PGI PDO	20	80
13	YES	ISO 9001 HACCP	YES	10	8,5	5	YES	PGI PDO	51	100
14	YES	HACCP	YES	8	100	100	YES	PGI PDO	12	100
15	YES	HACCP	NO	N.I.			YES	PGI PDO	25	N.I.
16	YES	HACCP	YES	13	100	0	YES	PDO	44 for PDO	100
17	YES	HACCPBRC FOOD	YES	3	50	N.I.	YES	PGI PDO	59 for PDO30 for IGT	100
18	YES	HACCP	YES	4	0	5	YES	PGI PDO	27	100
Total companies with certification	-	3	16	-	-	-	18	-	-	-
Year of certification (mean)	-	-	-	11.19	-	-	-	-	34.53	-
Turnover from certification (mean)	-	-	-	-	54.63	-	-	-	-	95.19

N.I.—Not Identified.

**Table 8:** Relevant factors in determining the price of wine (importance attributed on a Likert scale of 1 = not at all important to 5 = very important).

	Base Line				Premium Line			
	Business Costs	Brand Reputation	Price of Competitors	Brand Positioning	Business Costs	Brand Reputation	Price of Competitors	Brand Positioning
1	N.I.	N.I.	N.I.	N.I.	N.I.	N.I.	N.I.	N.I.
2	4	1	3	1	4	1	3	1
3	5	4	4	4	4	5	1	5
4	5	4	4	4	3	5	2	5
5	5	4	5	2	5	4	5	3
6	5	2	4	2	5	3	4	3
7	4	1	1	1	2	3	1	3
8	4	4	4	4	2	4	2	4
9	4	3	5	3	3	5	3	5
10	4	2	1	2	4	2	1	2
11	N.I.	N.I.	N.I.	N.I.	N.I.	N.I.	N.I.	N.I.
12	5	4	4	5	4	5	4	5
13	4	3	1	4	4	4	1	5
14	3	4	4	4	3	4	4	4
15	3	3	4	3	3	5	1	5
16	3	4	1	4	4	4	1	4
17	5	4	4	4	5	5	3	4
18	4	2	1	3	4	2	1	3
Mean	4.19	3.06	3.13	3.13	3.69	3.81	2.31	3.81
St. Dev.	0.750	1.124	1.544	1.204	0.946	1.276	1.401	1.223

N.I.—Not Identified.

**Table 9:** Communication tools.

Company	Social Media	Web Advertising	Web Marketing	TV	Radio	Press	Fairs and Events	Industry Magazines	Industry Guides	Newsletter	Corporate Annual Budget for Promotional Activities (€)
1	x		x				x				-
2	x		x				x		x		10/15,000 (100% Italy)
3	x		x				x	x	x		40,000 (100% Italy)
4	x		x				x	x	x		10,000(80% Italy; 20% Foreign)
5	x		x				x		x		2500 (100% Italy)
6	x		x				x	x	x		40,000 (100% Italy)
7	x		x			x	x		x		50,000 (100% Italy)
8	x	x	x			x	x	x			390,000 (66% Italy; 33% Foreign)
9	x		x				x	x			50,000 (70% Italy; 30% Foreign)
10	x	x	x			x	x		x		13,000 (50% Italy; 50% Foreign)
11	x	x	x			x	x				N,I,(100% Italy)
12	x		x		x	x	x	x			200,000 (90% Italy; 10% Foreign)
13	x		x			x	x	x	x		270,000 (80% Italy; 20% Foreign)
14	x	x	x						x		10,000 (70% Italy; 30% Foreign)
15	x		x				x	x			70,000 (90% Italy; 10% Foreign)
16	x		x			x	x	x			40,000 (30% Italy; 70% Foreign)
17	x	x	x		x		x				225,000 (100% Italy) Fairs (10% Italy; 90% Foreign)
18	x		x				x				25,000 (80% Italy; 20% Foreign) Fairs 100,000 (50% Italy; 50% Foreign)

**Table 10:** Strength, Weaknesses, Opportunities, and Threats (SWOT) analysis.

**Strenght**	**Weaknesses**
(i) Recognized and appreciated quality products (even with prizes) both in Italy and abroad (ii) Numerous certified organic wines (iii) Strong links with the territory and local traditions (iv) Entrepreneurial passion and stability in generational turnover (v) Ability to adapt to market changes (vi) Diversification of business models and openness to links with tourism	(i) Small company size, with few resources to invest (ii) Lack of clear and defined strategies on the part of companies (iii) Limits in management and marketing skills (iv) Strong attachment to the individual identity of the companies (v) Difficulty in accepting aggregative solutions, above all for the development of the markets, even though the need is felt (vi) Variety of wines and lack of a flagship wine
(i) Growing consumption of wine in foreign countries (ii) Popularity and tradition of Italian wine, appreciated all over the world (iii) Presence of agri-food productions and traditions complementary to the wine offer (iv) Territory rich in elements of values (culture, landscape, history, art, environment) (v) Region with strong elements of tourist attraction (sea + hinterland) (vi) Regional policies oriented towards the enhancement of the sector (vii) Presence of associative entities capable of promoting common initiatives (viii) Presence of organic district	(i) Increased costs (ii) Strong competition from Italian wines with similar territorial characteristics (iii) Region not strongly identified in the tourist attraction capacity (iv) Tendency to lose relevance of organic products
**Opportunities**	**Threats**

## Data Availability

The data presented in this study are available on request from the corresponding author. The data are not publicly available due to privacy.
